# Haploinsufficiency of *RPS14* in 5q− syndrome is associated with deregulation of ribosomal- and translation-related genes

**DOI:** 10.1111/j.1365-2141.2008.07178.x

**Published:** 2008-07

**Authors:** Andrea Pellagatti, Eva Hellström-Lindberg, Aristoteles Giagounidis, Janet Perry, Luca Malcovati, Matteo G Della Porta, Martin Jädersten, Sally Killick, Carrie Fidler, Mario Cazzola, James S Wainscoat, Jacqueline Boultwood

**Affiliations:** 1 LRF Molecular Haematology Unit, NDCLS, John Radcliffe HospitalOxford, UK; 2 Division of Hematology, Department of Medicine, Karolinska InstitutetStockholm, Sweden; 3 Medizinische Klinik II, St Johannes HospitalDuisburg, Germany; 4 Division of Hematology, University of Pavia Medical School, IRCCS Policlinico S. MatteoPavia, Italy; 5 Department of Haematology, Royal Bournemouth HospitalBournemouth, UK

**Keywords:** 5q− syndrome, *RPS14*, haploinsufficiency, microarray, ribosomes

## Abstract

We have previously demonstrated haploinsufficiency of the ribosomal gene *RPS14*, which is required for the maturation of 40S ribosomal subunits and maps to the commonly deleted region, in the 5q− syndrome. Patients with Diamond-Blackfan anaemia (DBA) show haploinsufficiency of the closely related ribosomal protein RPS19, and show a consequent downregulation of multiple ribosomal- and translation-related genes. By analogy with DBA, we have investigated the expression profiles of a large group of ribosomal- and translation-related genes in the CD34^+^ cells of 15 myelodysplastic syndrome (MDS) patients with 5q− syndrome, 18 MDS patients with refractory anaemia (RA) and a normal karyotype, and 17 healthy controls. In this three-way comparison, 55 of 579 ribosomal- and translation-related probe sets were found to be significantly differentially expressed, with approximately 90% of these showing lower expression levels in the 5q− syndrome patient group. Using hierarchical clustering, patients with the 5q− syndrome could be separated both from other patients with RA and healthy controls solely on the basis of the deregulated expression of ribosomal- and translation-related genes. Patients with the 5q− syndrome have a defect in the expression of genes involved in ribosome biogenesis and in the control of translation, suggesting that the 5q− syndrome represents a disorder of aberrant ribosome biogenesis.

The 5q− syndrome is the most distinct of all the myelodysplastic syndromes (MDS) (Boultwood *et al*, 1994a; Giagounidis *et al*, 2004). Van den Berghe *et al* (1974) first described the 5q− syndrome, noting the consistent association of the loss of the long arm of chromosome 5 [del(5q)] with the following haematological features: macrocytosis, anaemia, normal or high platelet count and hypolobulated megakaryocytes in the bone marrow. A female preponderance and a good prognosis have been widely reported in the 5q− syndrome (Boultwood *et al*, 1994a; Giagounidis *et al*, 2004). The 5q− syndrome is characterised by a clear genotype-phenotype relationship that is not found in other MDS and acute myeloid leukaemia (AML) characterised by chromosomal deletions. The 5q− syndrome is recognised as a distinct clinical entity according to the World Health Organization classification and is defined by a medullary blast count of <5% and the presence of the del(5q) as the sole karyotypic abnormality (Vardiman *et al*, 2002).

The del(5q) in the 5q− syndrome is considered to mark the location for a gene(s), the loss of which may affect important processes such as growth control and normal haematopoiesis (Boultwood *et al*, 1994a). Our group identified the commonly deleted region (CDR) or critical region of gene loss of the 5q− syndrome (Boultwood *et al*, 1994b; Jaju *et al*, 2000) and recently narrowed the CDR to the approximately 1·5 Mb interval at 5q32 flanked by D5S413 and the *GLRA1* gene (Boultwood *et al*, 2002). A transcription map of the CDR has been generated (Boultwood *et al*, 1997, 2000a, b) and the Ensembl gene prediction program was used for the complete genomic annotation of this region (Boultwood *et al*, 2002). We have shown that the CDR of the 5q− syndrome is gene rich (Boultwood *et al*, 2002) and have suggested that one or more of the 44 candidate genes mapping within this interval represents the gene or genes critical to the development of the 5q− syndrome (Boultwood *et al*, 2002). Several promising candidate genes have been identified including the tumour suppressor gene *SPARC*, and *RPS14*, a component of the 40S ribosomal subunit, and several microRNA genes (Boultwood *et al*, 2002, 2007). We performed mutation analysis of all 44 genes mapping to the CDR in a group of patients with the 5q− syndrome; no mutations were identified (unpublished data), supporting the proposal that haploinsufficiency (a gene dosage effect) of one or more of the genes mapping to the CDR is the pathogenetic basis of the 5q− syndrome.

We have recently demonstrated haploinsufficiency of the ribosomal gene *RPS14* in the haematopoietic stem cells (HSC) in patients with the 5q− syndrome (Boultwood *et al*, 2007) and have suggested that it represents a good candidate gene based on analogy with Diamond-Blackfan anaemia (DBA). The small-subunit protein Rps14, the yeast homolog of the bacterial S11 protein, directly binds helix 28 of 18S rRNA and is essential for the assembly of 40S ribosomal subunits (Larkin *et al*, 1987; Moritz *et al*, 1990). Importantly, haploinsufficiency of a closely related ribosomal protein, RPS19, also required for the maturation of 40S ribosomal subunits (Flygare *et al*, 2007), is one of the causative genes for DBA (Draptchinskaia *et al*, 1999). DBA is a broad developmental disease characterised by anaemia, bone marrow erythroblastopenia and an increased incidence of malignancy (Lipton *et al*, 2006). Mutations in *RPS19* are found in approximately 25% of DBA patients and lead to haploinsufficiency of *RPS19* (Flygare & Karlsson, 2007). It has recently been suggested that missense mutations in *RPS19* in DBA affect the capacity of the protein to be incorporated into preribosomes, thus blocking maturation of the pre40S particles (Gregory *et al*, 2007). Targeted degradation of the *RPS19* transcript, through retroviral expression of short hairpin RNAs (shRNAs) has been shown to block the proliferation and differentiation of erythroid progenitor cells in cultured human CD34^+^ cells (Ebert *et al*, 2005). Therefore, deficiency of RPS19 blocks proliferation of immature erythroid progenitor cells. Recently, the identification of a second DBA gene has established DBA as a ribosomal disorder because the affected gene (*RPS24*) encodes ribosomal protein S24 (Gazda *et al*, 2006a).

The anaemia in DBA and the 5q− syndrome is due to a failure of erythropoiesis and intriguingly both disorders show haploinsufficiency for ribosomal proteins, RPS19 and RPS14 respectively, required for the maturation of 40S ribosomal subunits. Most recently, Ebert *et al* (2008) have shown that the knock-down of *RPS14* in CD34^+^ cells using RNAi results in a block in erythroid differentiation (leading to erythroid cell apoptosis) with relative preservation of megakaryocyte differentiation, closely mirroring the defects observed in the 5q− syndrome. Moreover, forced expression of an *RPS14* cDNA in primary bone marrow cells from patients with the 5q− syndrome rescued the phenotype (Ebert *et al*, 2008). *RPS14* clearly represents a strong candidate gene for the 5q− syndrome (Boultwood *et al*, 2007; Ebert *et al*, 2008).

In RPS19-deficient DBA, the impaired 40S ribosomal subunit biogenesis suggests impaired translation as the mechanism that causes anaemia in DBA (Flygare & Karlsson, 2007). In accord with this hypothesis, global gene expression profiles in the haematopoietic progenitor cells of patients with DBA are characterised by downregulation of multiple ribosomal genes, as well as several genes which are required for translation initiation and elongation (Gazda *et al*, 2006b). Intriguingly, we have previously shown that MDS patients with the del(5q) show deregulation of genes involved in translation initiation when compared to MDS without the del(5q) (Pellagatti *et al*, 2006). Prompted by these observations, together with our recent demonstration of haploinsufficiency of *RPS14* in the 5q− syndrome, we investigated the expression levels of ribosomal genes and genes involved in translation initiation and elongation in the HSC of patients with the 5q− syndrome.

## Materials and methods

### Sample collection and cell separation

Fifteen patients with MDS 5q− syndrome, 18 MDS patients with refractory anaemia (RA) and a normal karyotype, and 17 healthy controls were included in the study. Classification of MDS patients was according to the French–American–British (FAB) criteria (Bennett *et al*, 1982). Patients with the 5q− syndrome had a del(5q) as the sole chromosomal abnormality and characteristic clinical morphological features (Giagounidis *et al*, 2004). The MDS patient samples were collected from several centres: Oxford and Bournemouth (United Kingdom), Duisburg (Germany), Stockholm (Sweden) and Pavia (Italy). The study was approved by the ethics committees (Oxford C00.196, Bournemouth 9991/03/E, Duisburg 2283/03, Stockholm 410/03, Pavia 26264/2002) and informed consent was obtained. Bone marrow samples were obtained and CD34^+^ cells isolated from MDS patients and healthy controls. Mononuclear cells were separated using Histopaque (Sigma-Aldrich, Gillingham, UK) density gradient centrifugation, labelled with CD34 MicroBeads, and then CD34^+^ cells were isolated using MACS magnetic cell separation columns (Miltenyi Biotec, Bergisch Gladbach, Germany) according to the manufacturer's recommendations. The purity of CD34^+^ cell preparations was evaluated with FACS and was ≥90%.

### Affymetrix experiments

Total RNA was extracted using TRIZOL (Invitrogen, Paisley, UK) following the manufacturer's protocol. The quality of the RNA samples was evaluated using Agilent Bioanalyzer 2100 (Agilent Technologies, Palo Alto, CA, USA). For each sample, 50 ng of total RNA were amplified and labelled with the Two-Cycle cDNA Synthesis and the Two-Cycle Target Labelling and Control Reagent packages (Affymetrix, Santa Clara, CA, USA). 10 μg of biotin-labelled fragmented cRNA was hybridised to GeneChip Human Genome U133 Plus 2.0 arrays (Affymetrix), covering over 47 000 transcripts representing 39 000 human genes. Hybridisation was performed at 45°C for 16 h in Hybridization Oven 640 (Affymetrix). Chips were washed and stained in a Fluidics Station 450 (Affymetrix) and scanned using a GeneChip Scanner 3000 (Affymetrix).

### Microarray data analysis

Cell intensity calculation and scaling was performed using GeneChip Operating Software (GCOS). Data analysis was performed using GeneSpring 7.3.1 (Agilent Technologies). The GCOS software was used to perform quality control after scaling the signal intensities of all arrays to a target of 100. The values obtained for scale factors, background levels, percentage of present calls, 3′/5′*GAPDH* ratio and intensities of spike hybridisation controls were within the acceptable range for all samples. Affymetrix CEL files were preprocessed using Robust MultiChip Analysis (RMA) (Irizarry *et al*, 2003). Hierarchical clustering was performed with GeneSpring software using Pearson correlation. The Database for Annotation, Visualization and Integrated Discovery (DAVID; http://david.abcc.ncifcrf.gov/) (Dennis *et al*, 2003) was used to identify enriched biological themes, in particular gene ontology terms.

### Real-time quantitative polymerase chain reaction (PCR)

The expression data for selected genes were validated using real-time quantitative PCR. The expression level of the *beta-2-microglobulin* gene (*B2M*) was used to normalise for differences in input cDNA. Predeveloped TaqMan Assays were used (Assays-on-Demand, Applied Biosystems, Foster City, CA, USA) and reactions were run on a LightCycler 480 Real-Time PCR System (Roche Diagnostics, Lewes, UK). Each sample was performed in triplicate and the expression ratios were calculated using the ΔΔC_T_ method (Livak & Schmittgen, 2001).

## Results

**Fig 1:**
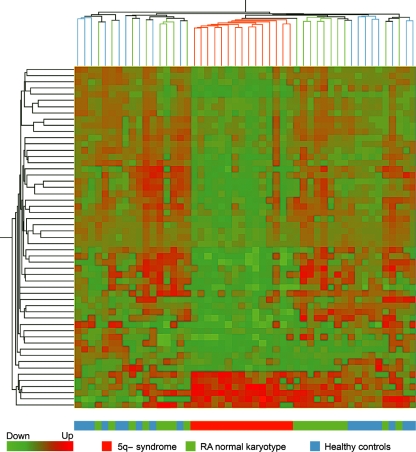
Hierarchical clustering of 55 differentially expressed genes between MDS patients with 5q− syndrome (red), MDS patients with RA and a normal karyotype (green), and healthy controls (blue). Each row represents a single Affy probe set and each column a separate CD34+ sample.

**Fig 2:**
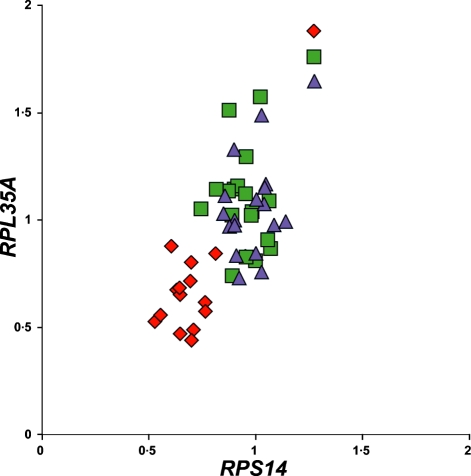
Scatterplot of the ratios for the genes *RPS14* and *RPL35A* in patients with the 5q− syndrome, patients with RA and a normal karyotype, and healthy controls.

The expression profiles of 579 probe sets for ribosomal- and translation-related genes (obtained from the GeneSpring software) were evaluated in the CD34^+^ cells obtained from 15 MDS patients with 5q− syndrome, 18 MDS patients with RA and a normal karyotype and 17 healthy controls. Of these 579 probe sets, 229 were for RPL (large subunit ribosomal protein) genes, 176 for RPS (small subunit ribosomal protein) genes, 149 for EIF (eukaryotic translation initiation factor) genes and 25 for EEF (eukaryotic translation elongation factor) genes. 55 of these 579 ribosomal- and translation-related probe sets were significantly differentially expressed (analysis of variance [anova] *P* < 0·01, Benjamini-Hochberg multiple testing correction) in the three-way comparison of the 5q− syndrome, MDS with RA and a normal karyotype, and healthy control groups, with four probe sets mapping to chromosome 5q (Table I). 49 of the 55 (89%) significantly differentially expressed probe sets showed lower expression levels in the 5q− syndrome patient group. Hierarchical clustering performed using these 55 probe sets (26 RPL, 15 RPS, 11 EIF, 3 EEF) grouped the patients with 5q− syndrome together, while no separation was observed between MDS patients with RA and a normal karyotype and healthy controls (Fig 1). A two-way scatterplot using the data for two of the most significant genes identified could effectively separate the patients with 5q− syndrome from the patients with RA and a normal karyotype and the healthy controls (Fig 2).

**Table I:** The significant differentially expressed probe sets between MDS patients with 5q− syndrome, MDS patients with RA and a normal karyotype, and healthy controls. Probe sets are ranked by decreasing *P*-value after adjustment for multiple testing. Genes in bold are common with Gazda *et al* (2006b). The full dataset is available as Supplementary Information (Table SI).

Probe set ID	Gene symbol	Map	Mean ratio 5q− syndrome	Mean ratio RA normal karyotype	Mean ratio healthy controls	Adjusted *P*-value
218007_s_at	*RPS27L*	15q22·2	1·93	1·34	0·95	3·25 × 10^−8^
214919_s_at	*EIF4EBP3*	5q31·3	0·53	1·17	1·01	4·00 × 10^−7^
208645_s_at	***RPS14***	5q31-q33	0·68	0·94	0·97	6·28 × 10^−6^
218339_at	*MRPL22*	5q33·1-q33·3	0·54	1·16	1·00	7·36 × 10^−6^
238026_at	*RPL35A*	3q29-qter	0·64	1·06	1·02	1·77 × 10^−5^
223015_at	*EIF2A*	3q25·1	0·62	0·77	0·96	9·10 × 10^−5^
210501_x_at	*EIF3K*	19q13·2	0·75	1·07	1·04	1·05 × 10^−4^
212039_x_at	*RPL3*	22q13	0·92	1·01	0·99	1·10 × 10^−4^
225541_at	*RPL22L1*	3q26·2	0·64	1·09	1·02	1·77 × 10^−4^
213223_at	***RPL28***	19q13·4	0·73	1·10	1·00	3·37 × 10^−4^
217719_at	*EIF3EIP*	22q	0·83	0·98	1·01	3·85 × 10^−4^
227708_at	*EEF1A1*	6q14·1	0·56	0·85	0·97	5·10 × 10^−4^
200005_at	*EIF3D*	22q13·1	0·81	1·10	1·10	5·14 × 10^−4^
203113_s_at	***EEF1D***	8q24·3	0·72	1·12	1·05	5·75 × 10^−4^
214317_x_at	*RPS9*	19q13·4	0·88	1·07	1·00	1·22 × 10^−3^
216588_at	*RPL7*	8q21·11	0·80	0·88	0·96	1·22 × 10^−3^
214042_s_at	*RPL22*	1p36·3-p36·2	0·79	0·88	0·98	1·64 × 10^−3^
221593_s_at	*RPL31*	2q11·2	0·59	1·06	0·97	1·64 × 10^−3^
236990_at	*EIF2AK3*	2p12	0·68	0·82	1·07	1·77 × 10^−3^
224767_at	*RPL37*	5p13	0·77	1·23	1·03	1·97 × 10^−3^
214097_at	*RPS21*	20q13·3	0·57	0·91	0·88	1·97 × 10^−3^
200074_s_at	*RPL14*	3p22-p21·2	0·81	1·03	1·02	2·18 × 10^−3^
212578_x_at	*RPS17*	15q	0·91	0·99	1·01	2·64 × 10^−3^
200819_s_at	*RPS15*	19p13·3	0·92	1·03	0·99	2·64 × 10^−3^
214271_x_at	*RPL12*	9q34	0·91	1·06	0·99	4·54 × 10^−3^
229590_at	*RPL13*	16q24·3	0·73	1·22	1·05	4·54 × 10^−3^
238448_at	*MRPL19*	2q11·1-q11·2	1·41	1·00	1·00	5·78 × 10^−3^
224330_s_at	*MRPL27*	17q21·3-q22	1·32	1·47	1·02	5·82 × 10^−3^
226190_at	*RPL4*	15q22	1·54	1·13	1·02	5·86 × 10^−3^
208697_s_at	*EIF3E*	8q22-q23	0·80	0·99	1·00	6·13 × 10^−3^
200715_x_at	*RPL13A*	19q13·3	0·76	1·01	0·99	6·42 × 10^−3^
211937_at	*EIF4B*	12q13·13	0·88	1·22	1·03	6·49 × 10^−3^
212537_x_at	*RPL17*	18q21	0·87	1·03	1·00	6·49 × 10^−3^
227722_at	*RPS23*	5q14·2	0·53	0·80	0·95	6·49 × 10^−3^
1556383_at	*RPS27*	1q21	0·83	0·84	1·05	7·08 × 10^−3^
202029_x_at	*RPL38*	17q23-q25	0·91	1·07	1·00	7·54 × 10^−3^
200094_s_at	*EEF2*	19pter-q12	0·83	1·08	1·02	7·54 × 10^−3^
224930_x_at	*RPL7A*	9q34	0·89	1·01	1·04	7·61 × 10^−3^

By repeating the three-way comparison using all 54 675 genes on the array, 467 genes were significantly differentially expressed between the three groups (anova*P* < 0·01, Benjamini-Hochberg multiple testing correction). The web-accessible DAVID program was used to identify enriched biological themes, particularly gene ontology terms, within this gene list (Table II).

**Table II:** Significantly enriched gene ontology terms within the list of 467 significantly differentially expressed genes in 5q− syndrome versus RA with a normal karyotype versus healthy controls.

Gene ontology category	Number of genes	Adj. *P*-value
Protein biosynthesis	40	2·2 × 10^−7^
Macromolecule biosynthesis	40	4·1 × 10^−6^
Biosynthesis	52	1·5 × 10^−4^
Protein metabolism	96	1·9 × 10^−4^
Cellular biosynthesis	47	3·3 × 10^−4^
Cellular macromolecule metabolism	90	3·8 × 10^−4^
Translation	16	5·3 × 10^−4^
Cellular protein metabolism	88	5·3 × 10^−4^

We compared our data with the results reported by Gazda *et al* (2006b), describing the defective expression of ribosomal protein genes in DBA. Three of our 55 significantly differentially expressed ribosomal- and translation-related genes (*RPL28, RPS14* and *EEF1D*) were in common with this paper (Table I), and similarly we found them downregulated in patients with the 5q− syndrome. In addition, in the comparison using all genes on the array, two of the 467 significantly differentially expressed genes identified (*TNFRSF10B* and *BAX*) were common to the results of Gazda *et al* (2006b), and similarly we found them upregulated in patients with the 5q− syndrome.

**Fig 3:**
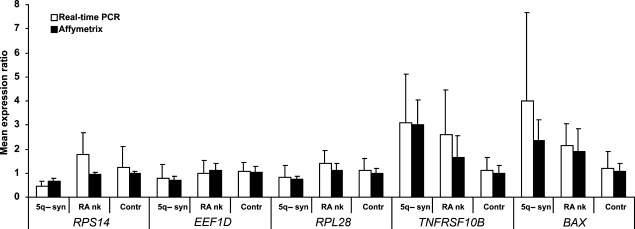
Comparison of the expression ratios obtained from real-time quantitative PCR (white bars) and Affymetrix experiments (black bars) for selected genes. The mean expression ratios and standard deviations are shown for each group. 5q− syn = 5q− syndrome, RA nk = RA with a normal karyotype, Contr = Healthy controls.

The expression levels of these five genes (*RPS14*, *EEF1D, RPL28, TNFRSF10B* and *BAX*), in common with Gazda *et al* (2006b), were validated using real-time quantitative PCR (Fig 3). Real-time quantitative PCR experiments confirmed the downregulation of *RPS14, EEF1D* and *RPL28* and the upregulation of *TNFRSF10B* and *BAX* in the 5q− syndrome patients.

## Discussion

Much progress has been made regarding the mapping of the CDR of the 5q− syndrome and the identification of candidate genes (Boultwood *et al*, 2002). One interesting gene that has been mapped to the CDR is the ribosomal gene *RPS14* (Boultwood *et al*, 2002)*,* encoding a protein required for the maturation of 40S ribosomal subunits (Larkin *et al*, 1987; Moritz *et al*, 1990). We have recently demonstrated haploinsufficiency of the ribosomal gene *RPS14* in the HSC of patients with the 5q− syndrome and have suggested that *RPS14* represents a good candidate gene for this disorder (Boultwood *et al*, 2007). Recently, Ebert *et al* (2008) provided compelling evidence demonstrating that *RPS14* is a causal gene for the 5q− syndrome. The analogy with DBA is very striking in that this disease is frequently caused by haploinsufficiency of another ribosomal protein, the RPS19 protein, and is characterised by a failure of erythropoiesis and an increased risk of leukaemic transformation (Lipton *et al*, 2006). The hypothesis under consideration in this study is that DBA and the 5q− syndrome share a related molecular basis in that they are both disorders of defective ribosomal biogenesis.

The expression profiles of a large group of ribosomal- and translation-related genes were determined in the CD34^+^ cells of 15 MDS patients with 5q− syndrome, 18 MDS patients with RA and a normal karyotype, and 17 healthy controls. 55 of these 579 ribosomal- and translation-related probe sets were significantly differentially expressed in the three-way comparison of the patient/control groups with approximately 90% of the significantly differentially expressed genes showing lower expression levels in the 5q− syndrome patient group. Hierarchical clustering performed using these 55 probe sets grouped the patients with 5q− syndrome together, while no separation was observed between MDS patients with RA and a normal karyotype and healthy controls. Thus patients with the 5q− syndrome can be separated both from other patients with RA and normal individuals solely on the basis of the deregulated expression of ribosomal- and translation-related genes.

Of the 55 significantly differentially expressed ribosomal-related probe sets in the HSC of the 5q− syndrome 26 represented RPL and 15 RPS. 35 out of these 41 probe sets showed downregulation in patients with the 5q− syndrome compared to healthy controls and patients with RA and a normal karyotype. Approximately half of these ribosomal genes showed a reduction in expression levels within the range 0·5–0·8 in patients with the 5q− syndrome, including *RPS14*, *RPS23*, *RPL28*, *RPL31* and *RPL22L1*. The findings of Gazda *et al* (2006b) indicate that some ribosomal protein genes are closely co-regulated in humans and that haploinsufficiency for RPS19 results in downregulation of the additional ribosomal protein genes in both haematopoietic progenitor and erythroid cells in DBA patients. We suggest that haploinsufficiency for RPS14 in the 5q− syndrome results in a similar downregulation of a group of ribosomal genes in the HSC of such patients.

The ratios for two of the most significant genes were plotted in a scatterplot to see if they alone could separate 5q− syndrome patients from patients with RA and a normal karyotype and healthy controls. The scatterplot obtained using the genes *RPL35A* and *RPS14* showed that the 5q− syndrome patients could be separated from patients with RA and a normal karyotype and healthy controls. This illustrates the diagnostic potential of ribosomal gene expression in the 5q− syndrome. It would be interesting to determine whether this diagnostic strategy could be transferred to peripheral blood leucocytes.

One ribosomal gene, *RPS27L*, showed upregulation by approximately twofold in patients with the 5q− syndrome compared to healthy controls. It has been recently shown that *RPS27L* is a p53-induced gene that promotes apoptosis (Lindstrom *et al*, 2007). Interestingly, a high level of apoptosis is found in patients with early MDS (Tehranchi *et al*, 2005), including the 5q− syndrome.

The HSC of patients with the 5q− syndrome also showed significant downregulation of many genes encoding proteins important for translation including the eukaryotic translation initiation factors *EIF2A* and *EIF3K* (*EIF3S12*)*,* the eukaryotic translation elongation factors *EEF1D* and *EEF1A1*. The fact that several significantly underexpressed genes encode proteins involved in translation suggests that this process is dysregulated in the HSC of patients with the 5q− syndrome. In support of this finding, we have previously reported that patients with MDS with a del(5q) show significant deregulation of genes involved in translational initiation when compared to MDS patients without the del(5q) (Pellagatti *et al*, 2006).

The data generated by this study in patients with the 5q− syndrome was compared with the data concerning deregulated ribosomal gene expression recently reported in RPS19-deficient DBA by Gazda *et al* (2006b) and, interestingly, the ribosomal- and translation-related genes *RPL28, RPS14* and *EEF1D* were found to be downregulated in both disorders. Moreover, several pro-apoptotic genes, including *TNFRSF10B* and *BAX* were upregulated in both disorders (Gazda *et al*, 2006b).

Next, in an analysis of the entire gene expression data sets, significantly deregulated biological processes were investigated in patients with the 5q− syndrome. The most significantly deregulated gene ontology category in patients with the 5q− syndrome compared to patients with RA and a normal karyotype and healthy controls was protein biosynthesis, with protein metabolism and translation also significantly deregulated. This data strongly supports the proposal that the HSC of patients with the 5q− syndrome are characterised by impaired protein biosynthesis and translation.

We have previously reported that the HSC of MDS patients with the del(5q), including patients with the 5q− syndrome, showed upregulation of histone genes and genes related to the actin cytoskeleton (Pellagatti *et al*, 2006; Boultwood *et al*, 2007). We suggest that these findings may be directly related to impaired translation in these cells. Intriguingly, superinduction of histone mRNAs has been reported when protein translation is inhibited (Sive *et al*, 1984). Moreover, it has recently been shown that improper organisation of the actin cytoskeleton affects protein synthesis at initiation (Gross & Kinzy, 2007). The eukaryotic translation elongation factor 1A (EEF1A) and other actin binding proteins are known to affect translation initiation through the actin cytoskeleton (Gross & Kinzy, 2007). The present study showed that *EEF1A* was downregulated in the HSC of patients with the 5q− syndrome by approximately 2-fold. Impaired protein translation in the HSC of patients with the 5q− syndrome may thus be the cause of the increased histone expression and deregulated actin cytoskeleton observed in these cells.

One important question is how a defect in ribosomal biogenesis and translation could lead to the development of a clonal haematological disorder. DBA is clearly associated with an increased risk of cancer (Lipton *et al*, 2006) and, as proposed for DBA, it may be that in the 5q− syndrome the secondary reduction of other ribosomal protein genes is a contributing factor. Indeed, there is increasing evidence that abnormalities in ribosome gene expression and biogenesis may play an important role in tumorigenesis. It is now recognised, for example, that certain ribosomal proteins have extraribosomal functions concerning the regulation of p53 and the cell cycle (Dai & Lu, 2004; Jin *et al*, 2004). Interestingly, it has recently been shown that many ribosomal genes are cancer causing genes in the zebrafish (Amsterdam *et al*, 2004). In an investigation of several hundred lines of zebrafish, each heterozygous for a recessive embryonic lethal mutation, 11 out of 12 lines with an elevated cancer incidence were heterozygous for a mutation in a different ribosomal protein gene. These findings are strong evidence that many ribosomal protein genes act as haploinsufficient tumour suppressors (Amsterdam *et al*, 2004). Intriguingly, several of these genes were amongst the group of ribosomal genes that were found to be downregulated in patients with the 5q− syndrome in this study, including *RPL7* and *RPL13*. Alternatively, it may be that haploinsufficiency of other genes localised within the CDR of the 5q− syndrome such as the tumour suppressor gene *SPARC* (Boultwood *et al*, 2007) play an important role in establishing clonal dominance in the 5q− syndrome.

The present study showed that patients with the 5q− syndrome have a defect in the expression of genes involved in ribosome biogenesis and in the control of translation. By analogy with DBA, we suggest that the deregulation observed in ribosomal gene expression and translation-related gene expression in the HSC of patients with the 5q− syndrome are secondary to RPS14 haploinsufficiency. These abnormalities may lead to impairment of ribosome biogenesis and subsequent reduction of protein translation capacity. The 5q− syndrome is characterised by a macrocytic anaemia and this ribosomal abnormality may be particularly crucial for developing erythroid cells, whose survival and division require large amounts of protein synthesis. These data support the hypothesis that the 5q− syndrome represents a disorder of impaired ribosomal biogenesis.

## Supplementary material

The following supplementary material is available for this article online:

Table S1 Spreadsheet containing the expression ratios of all 579 probe sets for ribosomal- and translation-related genes, for all the patients and controls included in the study.

The material is available as part of the online article from: http://www.blackwell-synergy.com/doi/abs/10.1111/j.1365-2141.2008.07178.x

(This link will take you to the article abstract).
