# Supplementary material for: Haploinsufficiency of RPS14 in 5q− syndrome is associated with deregulation of ribosomal- and translation-related genes
Source: Br J Haematol. 2008 Jul;142(1):57–64. doi: 10.1111/j.1365-2141.2008.07178.x (PMC2440427; doi:10.1111/j.1365-2141.2008.07178.x)
Supplement: Table S1 — Spreadsheet containing the expression ratios of all 579 probe sets for ribosomal- and translation-related genes, for all the patients and controls included in the study. [file bjh0142-0057-s1.doc]

**Table SI.** Spreadsheet containing the expression ratios of all 579 probe sets for ribosomal- and translation-related genes, for all the patients and controls included in the study.
